# The Role of Intraarterial Chemotherapy in the Management of Retinoblastoma

**DOI:** 10.1155/2020/3638410

**Published:** 2020-01-22

**Authors:** Aleksandra Pekacka

**Affiliations:** Institute of Ophthalmology, University College London, 11-43 Bath Street, London EC1V 9EL, UK

## Abstract

*Introduction*. Retinoblastoma is the most common primary intraocular neoplasm in children. With the advances in medicine, the armamentarium of available treatment modalities has grown. Intraarterial chemotherapy is a relatively new treatment method with promising outcomes. The purpose of this literature review is to evaluate its role in the management of retinoblastoma.

**Methods:**

A systematic online search was conducted using Ovid Embase and Ovid Medline. The final results included 23 studies. The studies were published between 2011 and 2019. The studies evaluated the technical success rate of IAC, globe salvage rate, and ocular and systemic complications, as well as the occurrence of deaths, metastasis, and secondary neoplasms. In total, 1827 eyes with retinoblastoma were analysed. The follow-up was between 0 and 252 months.

**Results:**

Overall globe retention rate ranged from 30% to 100%. Sixteen out of 23 studies reported ocular salvage between 60 and 80%. Eyelid oedema and erythema were the most commonly reported ocular complications following IAC. The most common systemic complications included nausea, vomiting, and neutropenia. Metastases and deaths were reported in 6 out of 23 studies. Three studies reported the development of secondary neoplasms. The technical success rate of IAC procedure ranged from 91% to 100%. *Discussion*. The studies have shown that IAC is a safe and effective treatment for advanced retinoblastoma, especially group D. It allows to save the globe without compromising patients' survival. Local and systemic complications are acceptable. The role of IAC in less advanced tumours is yet to be established. Future work should focus on conducting larger prospective studies with longer follow-up. Multiple novel therapies for the management of retinoblastoma are currently being tested, including angiogenic inhibitors and targeted agents. The results seem to be promising. Future advances require a further in-depth understanding of unique genetics of retinoblastoma and complex interactions between tumour cells and their microenvironment.

## 1. Introduction

Retinoblastoma is the most common primary intraocular neoplasm in children. It represents 10–15% of cancers that develop in the first year of life [1] and 3% of all malignancies in children [2].

It was first mentioned in the literature by Peter Pawius of Amsterdam in 1597 [2]. Over the years, researchers and physicians had different views regarding the origin of the tumour. In 1809, Scottish surgeon James Wardrop concluded that the tumour arose from the retina [2]. The name “retinoblastoma” was suggested by Verhoeff in the 1920s who stated that tumour cells histologically appeared like undifferentiated cells found in the embryonal retina called retinoblasts [3]. This terminology was introduced by the American Ophthalmological Society in 1926 [3].

Retinoblastoma leads to death if untreated; however, with current treatment modalities the survival rate reaches above 95% in the developed countries [1]. Developing world is less successful in curing the disease. Survival rates range from 40% in lower-income countries to 79% in upper-middle-income countries [4].

This review article focuses specifically on the role of intraarterial chemotherapy in the management of retinoblastoma. As IAC is a reality nowadays, there are significant amount of data supporting very good outcomes achieved with this treatment modality.

Intraarterial chemotherapy in retinoblastoma first emerged in 1958 when Reese injected triethylene melamine into the internal carotid artery [5]. Unfortunately, systemic toxicity associated with this procedure was too high; therefore, it was discontinued [5]. In 1998, Yamane et al. introduced for the first time “selective ophthalmic artery infusion” [6]. In this method, a microballoon catheter was inserted into the internal carotid artery distally to the ostium of the ophthalmic artery via a transfemoral approach [5]. The microballoon was used to occlude the internal carotid artery. Following the occlusion, melphalan was injected near the ostium of the ophthalmic artery [5]. The pioneers of this method reported 97.5% technical success rate [6]. In 2006, Gobin et al. introduced direct catheterization of the ophthalmic artery using a guide wire and called it “superselective intraophthalmic artery chemotherapy” [7]. The evolution of intraarterial chemotherapy contributed to increased safety and efficacy of this treatment method. Currently, it is widely used as primary and secondary therapy for intraocular retinoblastoma [5]. Moreover, it has been shown to be more effective in saving the globe in group D and E retinoblastoma compared to systemic chemotherapy [5]. It has also minimal systemic toxicity and allows for shorter treatment duration [5].

## 2. Disease Background

### 2.1. Genetics

Retinoblastoma develops due to germline or somatic mutations of both alleles of the RB1 gene which is recognized as a tumour suppressor gene [2]. RB1 is found in the long arm of chromosome 13 [2]. According to Knudson's two-hit hypothesis dated 1971, two consecutive mutations have to occur in order for malignant transformation to follow [2]. Two forms of retinoblastoma are distinguished, heritable and nonheritable. In heritable retinoblastoma, the first mutation known as the “first hit” occurs in the germ cell; therefore, the mutation is present in every cell of the body [8]. This makes the patient more prone to develop secondary cancers such as pineoblastoma, osteosarcoma, soft tissue sarcomas, and malignant melanoma [1, 2, 8]. However, the second mutation is needed to cause retinoblastoma [2]. This “second hit” affects retinal cells during their development [2]. Nonheritable retinoblastoma is associated with two somatic mutations. Both occur in the single retinal cell giving rise to tumour formation [2].

Nonheritable retinoblastoma is more common than the heritable form and accounts for 60–70% of all retinoblastomas [2]. The remaining 30–40% is heritable tumours [2]. It has to be emphasized that among germline mutations only a quarter are familial and characterised by an autosomal dominant pattern of inheritance while the rest are de novo nonfamilial mutations [8]. Germline tumours are characterised by incomplete penetrance meaning that approximately 90% of mutation carriers develop malignancy [1]. Patients with heritable retinoblastoma tend to have a bilateral, multifocal disease, positive family history, and known germline mutation [1]. In contrast, nonheritable retinoblastoma is usually unilateral and unifocal and with a negative family history [1].

Some of the tumours are caused by loss of the long arm of chromosome 13 [9]. This is known as RB1 gene deletion syndrome and apart from retinoblastoma it is characterised by dysmorphia and developmental delay [9].

### 2.2. Epidemiology

Retinoblastoma is a rare childhood cancer with the incidence of 1 in every 15,000 to 20,000 live births [10]. It constitutes 3% of all childhood cancers [11]. It is equally common in both genders and affects all races [1]. The vast majority of patients (95%) are diagnosed before they reach the age of five years [1]. Children with bilateral disease are diagnosed earlier than those with unilateral disease, and the average age at diagnosis is 12 months and 24 months, respectively [1].

### 2.3. Pathophysiology

Development of retinoblastoma is associated with mutations of both alleles of the RB1 gene [1]. These mutations cause gene inactivation and as a result, Rb protein, which acts as a tumour suppressor, is not produced [1]. It is thought that the cone precursor cell is the cell of origin in retinoblastoma [1].

Interestingly, it appears that RB1 gene mutation is not the only an event that can initiate retinoblastoma formation. According to Rushlow et al. [12], amplification of MYCN promotes uncontrolled cell proliferation and may initiate tumorigenesis even in the absence of RB1 mutations [12].

### 2.4. Natural History

If left untreated, retinoblastoma leads to death. Most commonly the tumour directly infiltrates the central nervous system through the optic nerve or invades the sclera and the orbit through the choroid [1]. Hematogenous spread to the lungs, bone, liver, or brain is also possible [1]. Lymphatic dissemination occurs when the tumour invades the conjunctiva, eyelids, or extraocular tissues [1]. It should be mentioned that in patients with heritable retinoblastoma, the secondary cancers are the main cause of death, not the primary tumour itself [1].

### 2.5. Clinical Presentation

The most common presenting sign of retinoblastoma is white pupillary reflex called leukocoria [10]. Strabismus appears to be the second most common feature and occurs when the tumour involves the central vision [10]. Other presentations of retinoblastoma include nystagmus, decreased vision, and ocular inflammation [1, 10]. Less commonly, the following presentations might be encountered: rubeosis iridis, vitreous haemorrhage, spontaneous hyphaema, glaucoma, anisocoria, orbital cellulitis, proptosis, painful eye, and fever [1, 10]. In case of metastatic disease, children might present with systemic symptoms such as weight loss, headache, nausea, vomiting, and neurological abnormalities on examination [1].

Retinoblastoma is a unifocal or multifocal, well-demarcated retinal tumour with or without tumour seeding [10]. Initially, it is translucent; however, as it grows it becomes opaque and white or cream in colour with dilated feeder vessels [2]. Small tumours are intraretinal [2]. As the disease progresses, retinoblastoma may show one of the three growth patterns: exophytic, endophytic, and diffuse infiltration [1]. The exophytic growth pattern is characterised by the tumour originating from the outer retina and extending away from the vitreous cavity and towards the subretinal space which is associated with subretinal seeds [1]. In the endophytic growth pattern, the tumour forms in the inner retinal layers and grows towards the vitreous cavity which leads to vitreous seeds [1]. Sometimes retinoblastoma may exhibit a combination of exophytic and endophytic growth patterns [2]. Diffuse retinal infiltration is a rare growth pattern, present in only 2% of cases, where the tumour remains flat and grows within the retina [1]. This leads to retinal ischaemia and provokes neovascularization [1].

Some patients may present with trilateral retinoblastoma which consists of unilateral or bilateral retinoblastoma and intracranial tumour, mostly arising in the pineal gland [1]. They are more common in patients with heritable disease than in nonheritable cases, 5% and less than 0.5%, respectively [1].

### 2.6. Classification

**Figure 1:**
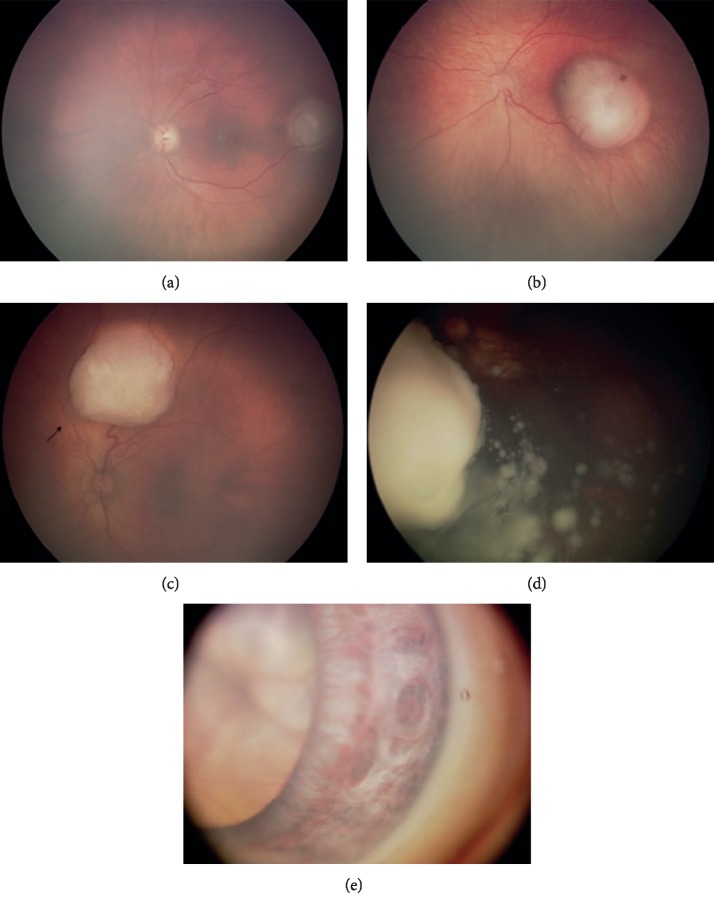
Retinoblastoma tumour grouping according to the International Intraocular Retinoblastoma 152 Classification. Group A: Tumour confined to the retina, >3 mm away from the macula (a); Group B: 153 tumour confined to the retina, at the macular region (b); Group C: local seeding (arrow) (c); Group D: 154 diffuse vitreous seeding (d); and Group E: iris neovascularization of the iris and a large tumour seen 155 behind the crystalline lens (e) reproduced from Ido Didi Fabian and Mandeep S. Sagoo 2019, [under the 156 Creative Commons Attribution 4.0 International License] [13].

The International Classification of Intraocular Retinoblastoma (ICIRB) is a system used worldwide to classify the disease according to its extent and the likelihood of globe salvage [10]. Historically, the Reese–Ellsworth classification system was used; however, it is now out of date and does not have application in clinical practice [1].  Table 1 presents the International Classification of Intraocular Retinoblastoma (see Figure 1 for example photographs of different stages of retinoblastoma according to ICIRB).  Table 2 shows the Reese–Ellsworth classification for retinoblastoma.

### 2.7. Differential Diagnosis

**Figure 2:**
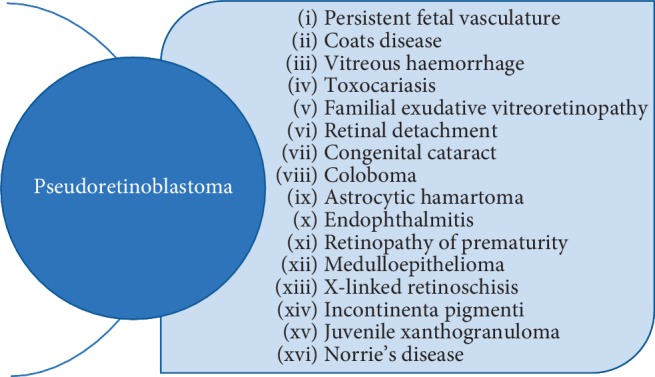
Differential diagnosis of retinoblastoma [2].

Since the most common presenting feature of retinoblastoma is leukocoria, other conditions that produce this sign have to be excluded [1, 11]. Possible causes for pseudoretinoblastoma are listed in Figure 2.

### 2.8. Investigations

**Figure 3:**
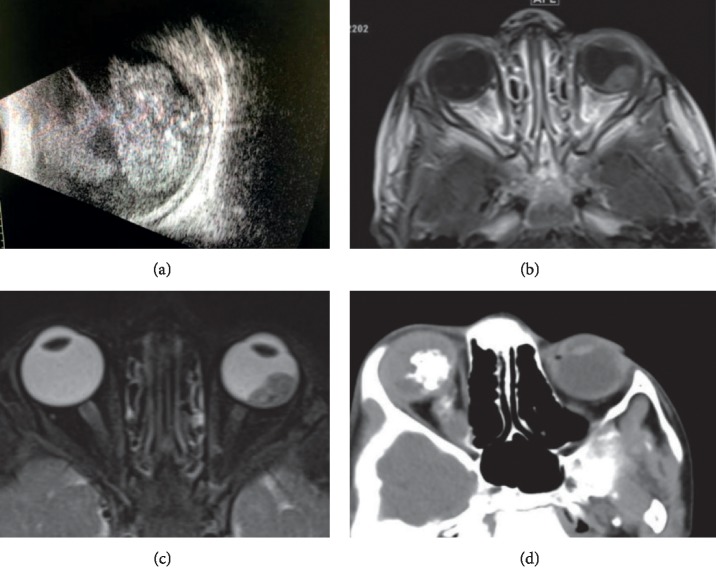
Different imaging modalities used to visualise retinoblastoma [15]. (a) B scan of an eye with diffuse pattern retinoblastoma showing areas of calcification (see left) reproduced from Fahad Albader and Dalal Fatani 2019, (under the Creative Commons Attribution 3.0 License). (b) MRI T1-weighted image with contrast, faintly enhanced retrolental mass at left globe corresponding to retinoblastoma tumor core [see left] reproduced from Fahad Albader and Dalal Fatani 2019, [under the Creative Commons Attribution 3.0 License]. (c) MRI T2-weighted fat saturated image of the orbits showing low signal of retrolental mass corresponding to retinoblastoma tumor at the left globe with mild reduction of size of globe (see left) reproduced from Fahad Albader and Dalal Fatani 2019, (under the Creative Commons Attribution 3.0 License). (d) CT (soft tissue window image) showing right globe hyperdense vitreous, retrolental intraocular solid mass with dystrophic calcification and proximal calcified optic nerve local invasion (see left) reproduced from Fahad Albader and Dalal Fatani 2019, [under the Creative Commons Attribution 3.0 License].

The diagnosis of retinoblastoma is clinical as the biopsy is contraindicated due to the risk of tumour seeding [1]. However, various imaging modalities help to assess the extent of the disease [15].  Table 3 presents common investigations and possible findings.  Figure 3 illustrates different imaging modalities used to visualise retinoblastoma.

It has to be emphasized that complete physical examination has to be performed in all high-risk patients [11]. If there is an evidence of a metastatic disease, a full metastatic screen is needed including bone marrow aspiration and biopsy, lumbar puncture, and radionuclide bone scan [11].

Genetic testing is important for all patients with retinoblastoma in order to identify whether the disease is heritable or nonheritable [1]. Testing of peripheral white blood cells allows to detect the germline mutation in 90–95% of cases [1]. If germline mutation is found, the siblings and offspring of the affected patient also need molecular genetic testing and surveillance for tumour [1].

### 2.9. Treatment

The primary goal of retinoblastoma management is life salvage [2]. However, with more efficient treatment modalities and improved survival rates, greater emphasis is currently put on eye salvage, improving visual outcomes, and prevention of secondary neoplasms [2, 16]. A multidisciplinary approach is essential in managing patients with retinoblastoma [3]. The team looking after the patient should include various healthcare professionals such as a paediatric ophthalmologist, paediatric oncologist, radiation oncologist, clinical geneticist, ocular oncologist, low-vision specialist, and nutritionist, to name a few [16]. The management of retinoblastoma has to be tailored to the individual patient and based on the stage of the disease [3]. Treatment modalities for the intraocular disease include focal therapy (cryotherapy, laser photocoagulation, and thermotherapy), chemotherapy (local and systemic), plaque brachytherapy, external beam radiotherapy, and enucleation [16]. It should be noted that external beam radiotherapy is no longer used as a first-line treatment due to associated complications [2].

### 2.10. Intraarterial Chemotherapy

It needs to be emphasized that intraarterial chemotherapy is an invasive procedure and has to be used with caution by an experienced multidisciplinary team [5].

IAC is performed under general anaesthesia on an outpatient basis [5]. Prior to the procedure, intravenous heparin (50–100 IU/kg body weight) is administered to attain anticoagulation [5]. Topical phenylephrine is applied on the forehead and intranasally to limit the chemotherapy flow [5]. The femoral artery on the side of the tumour is catheterized, and the catheter is advanced under fluoroscopy guidance [5]. It is essential to identify the anatomical variation of the ophthalmic artery while performing IAC. In 7% of cases, the ophthalmic artery is a branch of the middle meningeal artery; therefore, catheterization is achieved through the middle meningeal artery [5].

Once an angiogram confirms that the catheter is placed at the ophthalmic artery ostium, chemotherapeutics diluted in 30 ml of normal saline are infused over 30 minutes in a pulsatile fashion to ensure that the drug is evenly distributed [5]. It is vital to maintain the catheter's stability in order to minimise exposure to fluoroscopy radiation. At the end of the procedure, another angiogram is performed to exclude a thromboembolic event in the ophthalmic artery and to assess brain vascularization integrity [5]. The microcatheter and guide wire are removed and the femoral artery is manually compressed for 15 minutes to ensure hemostasis [5]. Patients are observed for 4–6 hours following the procedure, and if there are no concerns, they can be discharged home [5]. Between 7 and 10 days after IAC, all patients should have a complete blood count to assess for neutropenia [5]. Some teams also recommend oral aspirin for 2 weeks after procedure [5].

Chemotherapeutic agents administered intraarterially include melphalan, topotecan, and carboplatin. Inomata and Kaneko found in their study that melphalan appears to be the most potent 238 drug for retinoblastoma [18].

Standardized treatment protocol specifying drugs and dosage for intraarterial chemotherapy has not been established yet [5]. However, it appears that melphalan is the most commonly used drug among all agents [5]. Administration of intraarterial chemotherapy every 4 weeks for 3 sessions seems to be the most common approach [5]. Intraarterial chemotherapy is generally avoided in neonates and infants younger than 6 months due to difficulties in femoral artery cannulation [5].

Table 4 summarises types, doses, and indications for relevant intraarterial chemotherapeutic agents.

**Figure 4:**
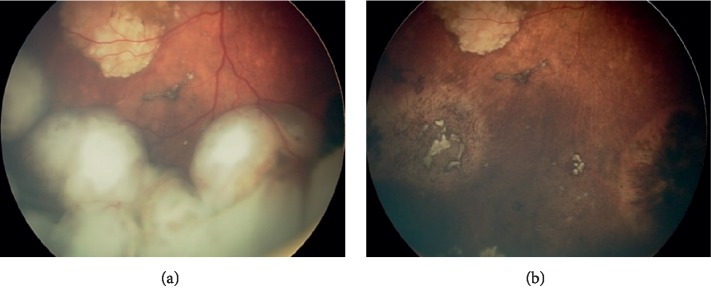
Intraocular retinoblastoma before and after treatement with intraarterial chemotherapy [18]. (a) Fundoscopic exam revealing an intraocular retinoblastoma before treatment with intra-arterial chemotherapy [see left] reproduced from Mario Zanaty et al. 2014, [under the Creative Commons Attribution 3.0 License]. (b) Fundoscopic exam revealing the same patient with intraocular retinoblastoma after treatment with intra-arterial chemotherapy [see left] reproduced from Mario Zanaty et al. 2014, [under the Creative Commons Attribution 3.0 License].

Intraarterial chemotherapy is indicated as a first-line treatment in unilateral retinoblastoma (group B, C, and D) and bilateral retinoblastoma (group D and E) [5]. However, it is common that some clinicians prefer systemic chemotherapy in patients with bilateral disease to avoid unpredictable vascular toxicity of IAC and ensure chemoprotection against metastasis and trilateral retinoblastoma [5]. IAC is used as a secondary therapy in recurrent/persistent tumours or recurrent/persistent subretinal seeds [5].  Figure 4 demonstrates an example photograph of group D retinoblastoma before and after IAC treatment.

**Figure 5:**
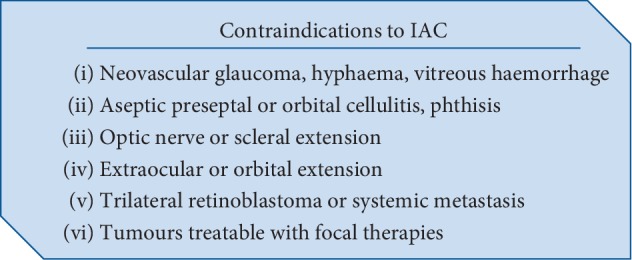
Contraindications to IAC [5].

It has to be emphasized that intraarterial chemotherapy cannot always be performed due to various contraindications listed in Figure 5.

As intraarterial chemotherapy is associated with exposure to fluoroscopy radiation, it is vital to consider its dosimetric impact on internal organs. Guasti et al. in their recent study proposed a method to estimate doses of radiation absorbed by internal organs which may help clinicians in planning their treatment [19].

## 3. Materials and Methods

A systematic online search was conducted on Ovid Embase (data published between 1947 and May 2019) and Ovid Medline (data published between 1946 and May 2019). Key concepts identified for this topic were “retinoblastoma” and “intraarterial chemotherapy.” Medical Subject Heading and Free Text Searches were used to conduct the searches. Apart from the keywords, synonymous terms and truncations were used. Boolean operators “OR” and “AND” were applied to merge the search lines. Retrieved references were exported to the EndNote file. Duplicate records were removed. Irrelevant articles were excluded based on the title, abstract, and if they did not meet the inclusion criteria. The final results were narrowed down to 23 studies.

### 3.1. Inclusion Criteria

- Clinical trials, reviews, and case series assessing the role of IAC for the management of retinoblastoma
- Studies published within the last 10 years
- Papers written in English
- Human-based studies

### 3.2. Exclusion Criteria

- Papers that did not assess the role of IAC for the management of retinoblastoma
- Studies published earlier than within the last 10 years
- Papers written in other languages than English
- Animal-based studies

## 4. Results

This literature review includes twenty-three studies. Majority of them are retrospective case series except for five prospective studies and one retrospective comparative study. Baseline characteristics of all studies can be found in Supplementary [Supplementary-material supplementary-material-1]. Detailed treatment outcomes, complications, and duration of follow-up are listed in Supplementary [Supplementary-material supplementary-material-1].

### 4.1. Globe Salvage

Gobin et al. found that the overall globe salvage rate with IAC used either as primary or secondary treatment was 80% including 100% globe salvage in Reese–Ellsworth groups I–IV and 77% in group V [7]. In the study by Suzuki et al., the overall ocular salvage was 60% [20]. Analysis of the individual groups revealed 100% eye survival in group A, 88% in group B, 65% in group C, 45% in group D, and 30% in group E [20]. Shields et al. in their study showed that 67% of eyes with retinoblastoma were saved [21]. This included 100% of group B and C eyes, 94% of group D eyes, and 36% of group E cases [21]. The globe salvage rate for eyes that received primary and secondary IAC was 72% and 62%, respectively [21]. In the study conducted by Abramson et al., 79% of eyes were salvaged including 85% of those that were treatment naive and 74% of those that received secondary IAC [22]. The overall globe salvage rate seems to be similar to the findings reported by Gobin et al. Swiss study by Munier et al. was the only comparative study found. It compared first-line IAC with first-line IVC in unilateral group D retinoblastoma [23]. None of the eyes treated primarily with IAC underwent enucleation; therefore, the globe salvage rate was 100% [23]. The globe was retained in 57% of eyes that received IVC as primary treatment [23]. The study by Francis et al. was characterised by the largest sample size including 436 eyes [24]. Authors reported that the overall globe salvage rate was 92% [24]. Interestingly, Hua et al. in their Chinese study reported an overall globe salvage of 30% [25]. 42% of group D eyes and 21% of group E eyes were spared enucleation [25]. This seems to be a lower success rate compared to the previous studies. This discrepancy might be due to the fact that all eyes included in the study had previously failed other treatments; therefore, relative chemotherapy resistance can be assumed [25].

In summary, the overall globe retention rate ranges from 30% as reported by Hua et al. [25] to 100% as demonstrated by Munier et al. [23]. However, the majority of analysed case series (sixteen out of twenty-three) reported ocular salvage between 60 and 80%. These results suggest that IAC can be considered an effective treatment for advanced retinoblastoma when used either as primary or secondary therapy. Furthermore, it allows to avoid enucleation and minimizes systemic toxicity. Nevertheless, more prospective studies with a larger sample size and longer follow-up are required to better assess the efficacy of IAC for the management of retinoblastoma.

The overall globe salvage rate and globe salvage by groups across analysed studies are described in detail in Table 5.

### 4.2. Ocular Complications

Ocular complications are an important indicator of localized IAC toxicity. Gobin et al. in their study reported 10 cases of eyelid oedema and erythema, 4 cases of avascular retinopathy with total visual loss, and loss of eyelashes in 12 patients [7]. In a study of Suzuki et al., periocular swelling and erythema was observed in some cases (exact amount not specified), orbital inflammation occurred in 2 patients, and chorioretinal atrophy was noted in 2 cases [20]. Shields et al. confirmed that the most common ocular complication was eyelid oedema noted in 10 eyes [21]. Other adverse events included vitreous haemorrhage in 4 eyes, BRAO in 2 eyes, ophthalmic artery spasm in 4 eyes, ophthalmic artery occlusion in 3 cases, and phthisis bulbi in 1 case [21]. Abramson et al. observed 25 cases of eyelid oedema and erythema which was the most common ocular adverse event [22]. They also reported retinal/choroidal vascular occlusions in 6 eyes, phthisis bulbi in 5 eyes, vitreous haemorrhage in 4 eyes, optic nerve swelling in 3 eyes, cranial nerve palsy in 2 cases, and suprachoroidal haemorrhage in 1 eye [22]. In a study by Munier et al., retinal detachment was most commonly reported following IAC treatment (14 eyes) [23]. Other adverse events included retinopathy in 8 eyes, cataract in 6 eyes, eyelid oedema in 5 eyes, and vitreous haemorrhage in 1 eye [23]. According to Hua et al., chemosis was the most common ocular complication and was noted in 25 eyes [25]. The second most common was eyelid oedema in 12 eyes, and then ophthalmic artery spasm with reperfusion in 11 eyes [25]. Vitreous haemorrhage was observed in 7 eyes, subretinal haemorrhage in 9 eyes, and retinal vasculopathy in 6 eyes [25].

Overall, it appears that eyelid oedema and erythema was the most commonly reported ocular complication following IAC. It is essential to emphasize that the majority of the ocular adverse events had transient character and usually resolved within days to months. However, it should be noted that some of the case series reported permanent damage to the eyes such as avascular retinopathy with total visual loss observed by Gobin et al. [7]. The potential risk of other severe complications such as vascular occlusions, chorioretinal atrophy, retinal detachment, neovascular glaucoma, and cranial nerve palsies should not be neglected. Reddy et al. suggested in their recent study that adjusting the dose of the intraarterial chemotherapeutic agent according to the patient's age may significantly decrease ocular adverse events and still maintain excellent globe salvage rates [38].

**Figure 6:**
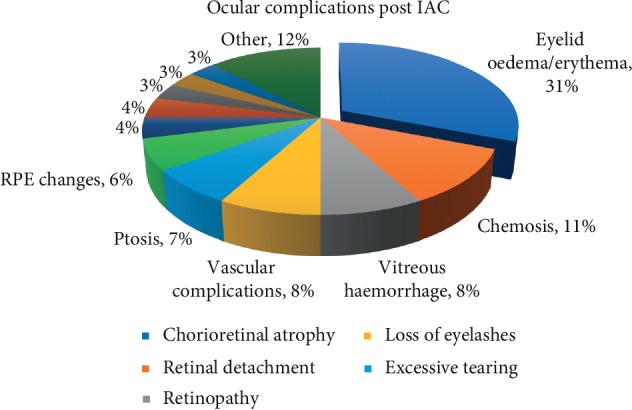
Ocular complications following IAC.

Figure 6 illustrates ocular complications following IAC and their percentage share based on the analysed studies.

### 4.3. Systemic/Extraocular Complications

Although the risk of systemic toxicity with intraarterial chemotherapy is significantly reduced compared to intravenous chemotherapy, it cannot be neglected. Systemic complications following IAC are mostly dependent on the dose of the chemotherapeutic agents and tend to be transient. It has to be appreciated that some of the adverse events are strictly associated with the catheterization procedure and include autonomic episodes and anaphylactic or anaphylactoid reactions.

Gobin et al. found that the most common systemic toxicity was neutropenia diagnosed in 29 patients [7]. Twenty-four patients developed bronchospasm during the procedure [7]. Other adverse events included forehead hyperemia in 14 patients, allergic reaction to iodinated contrast in 6 patients, superficial femoral artery occlusion in 1 patient, and neutropenic fever in 1 case [7]. According to Suzuki et al., transient vomiting 412 was the most common complaint following IAC and occurred in 58 patients [20]. Bradycardia was observed in 24 patients and bronchospasm in 1 patient [20]. Shields et al. found that forehead hyperemia occurred in 3 patients and scalp alopecia in 1 patient [21]. Apart from these, no other systemic adverse events were observed [21]. Interestingly, Akyuz et al. did not detect any systemic side effects in their study [33] which is in agreement with findings published by Parareda et al. [32]. Abramson et al. found that bronchospasm was the most common systemic complication during intraarterial drug delivery and occurred in 44 patients [22]. Neutropenia, developed by 39 patients, was the second most common side effect [22]. Other adverse events included allergy type reaction in 5 patients, thrombocytopenia in 4 patients, fever in 4 patients, cardiorespiratory side effects in 3 patients, injection site complications in 3 patients, and epistaxis in 1 case [22]. Munier et al. reported 4 cases of cardiorespiratory disturbances, 4 cases of nausea, 3 cases of neutropenia, 2 cases of internal carotid artery or ophthalmic artery spasm, and 1 case of frontal alopecia [23]. Hua et al. found that transient vomiting was commonly encountered following IAC and affected 17 patients [25]. Fourteen patients developed fever, and 8 patients developed transient myelosuppression [25]. It should be noted that some of the studies did not comment on the systemic complications such as Francis et al. [24] and Dalvin et al. [41].

**Figure 7:**
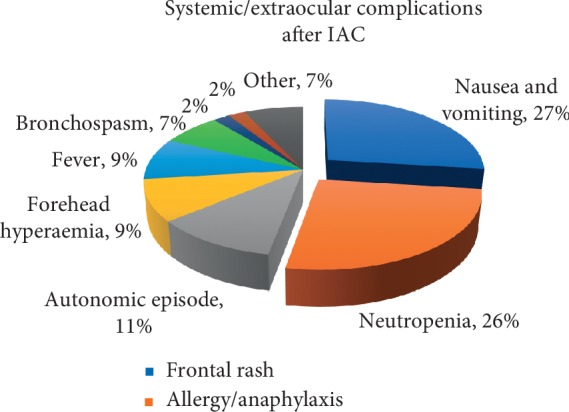
Systemic and extraocular complications following IAC.

Figure 7 illustrates systemic and extraocular complications following IAC and their percentage share based on the analysed studies.

To conclude, nausea and vomiting was the most common systemic adverse event across all studies accounting for 27% of reported systemic complications. The second most common complication was neutropenia. Autonomic episodes including bradycardia and hypotension during intraarterial drug delivery accounted for 11% of all systemic complications. True anaphylaxis or anaphylactoid reactions were rare although the risk of their occurrence exists. Across all case series, the anaphylactoid reaction was noted in 2 patients [28, 36] while anaphylaxis occurred only once following carboplatin administration [35]. It appears that IAC-related systemic side effects are mostly mild and transient; therefore, this treatment modality can be considered safe while performed by an experienced team of interventional neuroradiologists.

### 4.4. Metastasis, Death, and Secondary Cancers

Metastases, death, and secondary cancers are crucial events to consider when evaluating safety and efficacy of intraarterial chemotherapy for the management of retinoblastoma.

Metastases were reported in six out of twenty-three studies. In the study by Gobin et al., two patients developed metastatic disease [7]. Metastases were diagnosed 7 months and 9 months after enucleation for painful phthisis and disease recurrence, respectively [7]. Both children survived following aggressive treatment with systemic chemotherapy and focal therapies [7]. Suzuki et al. found in their study that 8 patients suffered from metastatic disease including 3 cases of brain metastases and 5 cases of multiple systemic metastases [20]. Unfortunately, none of them survived [20]. Akyuz et al. reported 2 cases of metastases [33]. Both patients belonged to the group that failed initial systemic chemotherapy and therefore received secondary IAC. Eventually, they had their eyes enucleated as they failed to respond to IAC. Following enucleation, both patients died as the disease progressed despite intensive treatment with radiotherapy and chemotherapy [33]. One patient developed brain metastases [33]. In the other patient, metastatic spread was not specified by the authors [33]. Ong et al. found that 3 patients progressed to metastatic disease [34]. All of them received IAC as a secondary treatment following disease recurrence or failure of previous therapy [34]. Two of them had bilateral retinoblastoma [34]. All of the patients developed metastasis to the central nervous system [34]. Two of them died even though their eyes had been enucleated and they had received aggressive systemic chemotherapy [34]. In the study of Abramson et al. three patients developed metastatic disease [22]. All of them belonged to the treatment-naive group, and all three survived following successful treatment [22]. Francis et al. reported that 5 patients progressed to metastatic disease including 2 patients that were treated in the preintravitreous chemotherapy era and 3 patients treated in the intravitreal era [24].

Deaths were reported in six out of twenty-three studies. Majority of them were discussed above. According to the findings of Suzuki et al., 12 patients died during the follow-up period [20]. Eight deaths were due to metastatic disease as mentioned before. The remaining 4 patients died of secondary AML, osteosarcoma, pinealoblastoma, and AML recurrence [20]. Abramson et al. reported 1 death in their study which was due to second nonocular neoplasm (pinealoblastoma) [22]. Francis et al. described 6 cases of death, four due to trilateral disease, one due to metastatic retinoblastoma, and one due to nonaccidental injury [24]. In the study by Funes et al., two deaths were reported [39]. Both patients suffered from bilateral retinoblastoma and died from extraocular spread of the disease after parental refusal to proceed with enucleation [39].

Secondary neoplasms were reported in three out of twenty-three studies. Suzuki et al. found that 11 patients developed 12 secondary neoplasms [20]. All of the patients had been previously treated with external beam radiotherapy [20]. Two patients suffered from unilateral retinoblastoma and 9 patients had bilateral disease [20]. Eight neoplasms were diagnosed within the radiation field, 3 outside of it and 1 was acute myeloid leukaemia [20]. These findings might suggest that secondary tumours are predominantly associated with the previous radiotherapy treatment and, to a lesser extent, with chemotherapy [20]. One of the patients developed 2 secondary neoplasms, preauricular rhabdomyosarcoma and orbital bone osteosarcoma [20]. Francis et al. reported 7 secondary neoplasms including 5 pineoblastomas, 1 orbital osteosarcoma and 1 neuroblastoma [24]. Funes et al. described 2 secondary malignancies [39]. One patient who was treated with systemic chemotherapy, external beam radiotherapy, and secondary IAC developed bone Ewing sarcoma out of the radiation field [39]. The other patient who received primary IAC for sporadic unilateral retinoblastoma developed a craniopharyngioma and underwent surgical removal of the tumour [39].

Table 6 presents the incidence of metastases, deaths, and secondary tumours in the analysed studies.

### 4.5. Technical Success

Technical success of the ophthalmic artery catheterization reported in the analysed studies ranged from 91% to 100%. Five studies reported a technical success rate of 100%. Eight studies did not comment on the achieved technical success. The lowest success rate of 91% was reported by Ong et al. [34]. The authors described 49 successful procedures out of 54 trials [34]. Five procedures failed because of decreased perfusion through the ophthalmic artery and contrast reflux into the internal carotid artery [34]. In 1 case, the ophthalmic artery was catheterized through the middle meningeal artery [34]. Gobin et al. found that catheterization was successful in 98.5% of procedures which is 255 out of 259 procedures [7]. Four attempts failed due to one of the following reasons: ophthalmic artery could not be selectively catheterized, middle meningeal artery did not provide collateral blood flow to the ophthalmic artery, or balloon technique could not be used due to tortuosity of the internal carotid artery or very young age of the patient [7]. Suzuki et al. used a balloon obstruction method to selectively access the ophthalmic artery [20]. They achieved a success rate of 98.8% which means 1452 out of 1469 procedures were successful [20]. Seventeen procedures failed because of the following reasons: femoral artery could not be punctured in 5 cases, the orifice of the ophthalmic artery could not be catheterized due to spasm in 4 cases, in 3 cases, ophthalmic artery was not imaged with the contrast, and 5 eyes had vascular anomalies [20]. Shields et al. in their study performed 198 catheterizations with a success rate of 99.5% [21]. One procedure was unsuccessful due to carotid artery spasm [21]. In one case, the balloon obstruction technique was used instead of superselective catheterization because the ophthalmic artery was too small for direct access [21].

In conclusion, it appears that ophthalmic artery catheterization has a high success rate if performed by skilled and experienced physicians. Most common causes of procedure failure include vasospasm, contrast reflux, and vascular anomalies.

### 4.6. Duration of Follow-Up

Mean duration of follow-up ranged from 8.6 months as reported by Peterson et al. [26] to 74 months as documented by Suzuki et al. [20]. However, it should be noted that in the majority of studies the mean follow-up period did not exceed 20 months. The short follow-up is one of the main limitations of the analysed case series. Future studies should aim for a long follow-up period in order to better assess long-term complications and incidence of secondary neoplasms.

### 4.7. Sample Size

Sample size plays a major role while evaluating the safety and efficacy of intraarterial chemotherapy for the management of retinoblastoma. Small sample size may significantly affect the reliability of the outcomes. Sample size in the analysed studies ranged from 9 eyes as in the study by Reddy et al. [38] to 436 eyes as reported by Francis et al. [24]. It has to be emphasized that the number of eyes was less than 40 in at least half of the analysed studies. The studies with the largest sample size were published by Francis et al. (436 eyes) [24] and Suzuki et al. (408 eyes) [20]. Reddy et al. [38] and Parareda et al. [32] included in their studies only 9 and 12 eyes, respectively. These differences have to be considered during the critical appraisal process.

Table 7 summarises sample size, technical success rate, and duration of follow-up across all analysed studies.

## 5. Future Treatment Options

**Figure 8:**
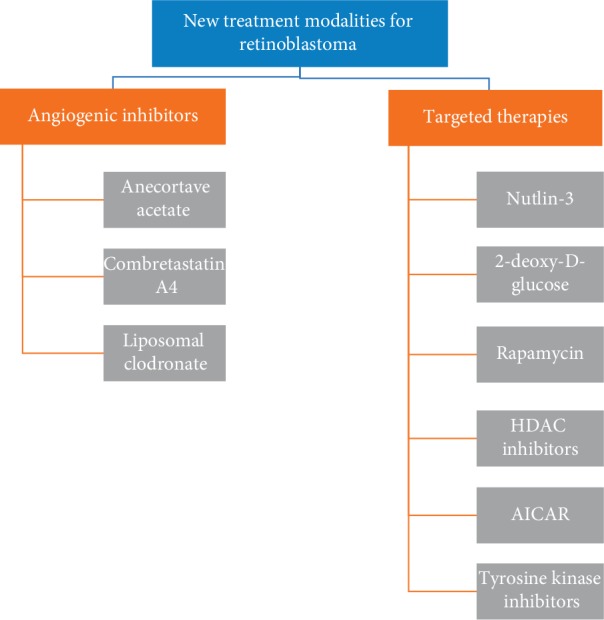
Summary of new treatment modalities for retinoblastoma [42–44].

Currently, research in retinoblastoma is focused on new targeted treatment approaches that maximize effectiveness and cause minimal toxicity. In order to achieve these goals, further understanding of complex genetics and interactions between tumour cells and their local microenvironment is required. It is crucial to emphasize the role of animal models in exploring retinoblastoma tumorigenesis and testing new therapies. New treatment modalities for retinoblastoma include angiogenic inhibitors and various targeted therapies (see Figure 8) [42–44].

### 5.1. Angiogenic Inhibitors

Tumour vasculature seems to be a potential therapeutic target in retinoblastoma treatment. However, it has to be noted that mature and immature blood vessels are targeted by different agents [42]. Furthermore, angiogenic inhibitors used alongside systemic or intraarterial chemotherapy might significantly reduce the amount of chemotherapeutics delivered to the tumour if they are administered prior to chemotherapy [42]. Therefore, the timing of the combined treatment has to be optimal [42].

Anecortave acetate and combretastatin A4 belong to the group of angiogenic inhibitors that target primarily immature blood vessels [42]. Murine retinoblastoma models were used to study these vascular-targeting agents. It was found that they induce apoptosis and cause minimal necrosis [43]. Following subconjunctival administration, both agents have a maximal effect on tumour vasculature at day 1 [42]. This effect persists up to 1 week [42].

Tumour-associated macrophages (TAMs) and matrix metalloproteinases (MMPs) play an important role in tumorigenesis [43]. MMPs are responsible for the degradation of the extracellular matrix and take part in angiogenesis, cell proliferation, and metastasis [43]. TAMs were found to increase the levels of MMPs [43]. Therefore, both TAMs and MMPs are potential therapeutic targets. Studies on murine retinoblastoma models have proven that anecortave acetate reduces the expression of matrix metalloproteinases and as a consequence inhibits tumour growth [42]. On the other hand, TAMs are targeted by liposomal clodronate which causes their depletion and in fact improves tumour control [43].

### 5.2. Targeted Therapies

#### 5.2.1. Nutlin-3

Tumour protein p53 is a tumour suppressor that activates apoptosis and prevents cancer formation. It has been found that the MDMX/MDM2 signalling pathway in retinoblastoma is overexpressed which leads to inactivation of p53 [42]. While p53 is inactive, tumour cells expand [42]. Understanding the interaction between MDMX/MDM2 and p53 allowed to introduce nutlin-3 which is a novel agent used as an inhibitor of the MDMX/MDM2-p53 pathway [43]. Nutlin-3 promotes apoptosis in murine models of retinoblastoma [42]. It is believed that nutlin-3 can be used in combination with chemotherapeutics. According to Brennan et al. [45], subconjunctival administration of nutlin-3 and topotecan in a mouse model showed a reduction of the tumour size of 82-fold. No side effects were observed. Nutlin-3 is currently in phase 1 clinical trial for the management of retinoblastoma [43].

#### 5.2.2. 2-Deoxy-D-Glucose

Advanced retinoblastoma tumours show a high level of hypoxia exceeding 20% according to Boutrid et al. [46]. Tumour cells switch to anaerobic glycolysis as a form of adaptation to hypoxic conditions [42]. Glycolytic inhibitors such as 2-deoxy-D-glucose can be used as a potential therapy to target hypoxic cells [42]. Boutrid et al. [46] showed that systemic treatment with 2-deoxy-D-glucose led to significant tumour reduction in murine models and decreased hypoxia from 20% to less than 1%. Piña et al. [47] achieved promising outcomes with periocular injection of 2-deoxy-D-glucose. It should also be noted that 2-deoxy-D-glucose might have a potential role in the treatment of other human cancers when used either alone or in combination with additional therapies [43]. Currently, there are ongoing phase 1 and 2 clinical trials with 2-deoxy-D-glucose for the treatment of gliomas, lung, breast, prostate, and head and neck cancers [48]. The results are promising, and research in these areas might contribute to the development of new management strategies in retinoblastoma [43].

#### 5.2.3. Rapamycin

The mTOR pathway plays a vital role in cell growth, proliferation, and metabolism [42]. It has been demonstrated that this pathway is dysregulated in retinoblastoma as hypoxic tumour cells gain control of the mTOR signalling and use it to promote their proliferation [43]. Therefore, the mTOR pathway is yet another therapeutic target. The mTOR inhibitors such as rapamycin have been investigated by Pina et al. [49]. The authors demonstrated that periocular injections of rapamycin led to tumour reduction, decreased hypoxia, and shrinkage of tumour vasculature [49]. In contrast to previously mentioned angiogenic inhibitors, rapamycin tends to target primarily mature blood vessels [42]. Outcomes achieved in animal models are promising; however, further studies are needed to assess the safety and efficacy of this novel therapy [42].

#### 5.2.4. HDAC Inhibitors (Histone Deacetylase Inhibitors)

Histone deacetylases are enzymes responsible for the regulation of transcription. Deacetylated histones force the chromatin to remain in a condensed state which prevents transcription of genes [50]. Apart from histones, histone deacetylases interact with many other proteins involved in the control of tumour development such as p53 [50]. It has been shown that dysregulation of these interactions has tumorigenic potential [50]. Therefore, HDAC inhibitors might be a potential novel therapy in the management of retinoblastoma. According to the study by Dalgard et al. [50], HDAC inhibitors have antitumour effect noticed both in vitro and in preclinical animal models. Based on these findings, the authors suggested that clinical trials with HDAC inhibitors should be considered [50].

#### 5.2.5. AICAR

AMPK (AMP-activated protein kinase) has been identified as a regulator of cell growth and proliferation [42]. Activation of AMPK promotes apoptosis; therefore, this signalling pathway might be used to reduce tumour expansion in retinoblastoma. Theodoropoulou et al. in their study tried to investigate a potential agent that could induce AMPK activation [51]. This agent is AICAR (5-aminoimidazole-4-carboxamide riboside) which has been shown to inhibit the growth of retinoblastoma cell lines in vitro [42].

#### 5.2.6. Tyrosine Kinase Inhibitors

Tyrosine kinases are important modulators of cell functions including proliferation, differentiation, and attachment [52]. They are targets for therapies against retinoblastoma and uveal melanoma [52]. Up to date, there are a few studies on the use of tyrosine kinase inhibitors against retinoblastoma. Available data demonstrated good outcomes achieved in vitro with the use of afatinib, erlotinib (both are EGFR inhibitors), and imatinib (PDGFRA and PDGFRB inhibitor) [53–55].

In summary, various novel therapies for the management of retinoblastoma are currently being tested in vitro and in suitable animal models. The results are promising, and some of the agents have entered the clinical trial phase. Future advances require further in-depth understanding of unique genetics of retinoblastoma and complex interactions between tumour cells and their microenvironment [42].

## 6. Discussion

Eyes with advanced retinoblastoma constitute a major therapeutic challenge. With advances in medicine, the treatment goal is no longer only avoidance of enucleation but also preservation of vision. Historically, eyes with advanced tumours required systemic chemotherapy or external beam radiotherapy to save the globe.

External beam radiotherapy is known to cause frequent and severe complications such as cataract formation, radiation retinopathy, and increased incidence of secondary neoplasms [7]. For these reasons, in the 1990s, intravenous chemotherapy was introduced as the primary treatment for advanced retinoblastoma in place of radiotherapy. Nevertheless, it should be noted that intravenous chemotherapy causes severe systemic toxicity such as vomiting, myelosuppression, infection, ototoxicity, nephrotoxicity, secondary leukaemia, and infertility [7]. Importantly, systemic chemotherapy on its own is often insufficient to completely destroy the tumour and local therapies are needed [7].

With the advent of intraarterial chemotherapy that uses small doses of the chemotherapeutic agent and delivers it directly into the tumour bed, these systemic complications can be significantly minimized. Interestingly, the Japanese were the pioneers in introducing this technique as cultural attitudes in Japan did not support enucleation as a treatment modality [7]. In 2004, a balloon technique was developed by Yamane et al. and Suzuki and Kaneko [6, 56]. In this method, a microballoon was used to occlude the internal carotid artery distally to the ophthalmic artery. It should be noted that this technique was not truly selective as the drug could easily access intracranial vasculature. In 2008, Abramson et al. introduced a superselective technique where melphalan was delivered directly into the ophthalmic artery with a microcatheter. Currently, ophthalmic artery chemosurgery has been implemented in more than 45 countries around the world [22].

The primary objective of this literature review was to evaluate the effectiveness and safety of intraarterial chemotherapy for the management of retinoblastoma. The literature search revealed a large number of case series on the intraarterial drug delivery in patients with retinoblastoma. The majority of them are retrospective case series.

Analysis of the 23 studies allows to conclude that they share similar limitations. The most common limitations include small sample size, short duration of follow-up, retrospective study design, use of concurrent adjuvant therapies, variation in the types of chemotherapeutic agents that were administered, and variation in the interval between IAC sessions [7]. Additionally, different approaches for establishing drug dosage were seen across all studies. Some of the clinicians used body weight or body surface area while others were guided by the patient's age and angioanatomy [7]. This confirms that there is an emerging need for establishing a uniform protocol including optimal single dose, interval, and cumulative dose [20]. Furthermore, nearly all studies did not report any visual acuity outcomes. The exception is Suzuki et al. who found that more than 50% of eyes without macular involvement retained a visual acuity >0.5 [20].

The parameters used to assess the efficacy and safety of intraarterial chemotherapy were globe salvage rate, the occurrence of metastatic disease, death, or secondary neoplasm, and the incidence of ocular and extraocular complications. All of the analysed case series are in agreement that IAC is safe and effective in the management of advanced intraocular retinoblastoma.

Peterson et al. emphasized that although intraarterial drug delivery does not come without its risks, it became a treatment of choice for refractory retinoblastoma in their center and allowed to achieve good outcomes [26].

Suzuki et al. found in their study that IAC is not associated with severe ocular adverse events if performed up to 10 rounds [20]. After the 10th cycle, there is an increased risk of severe ocular complications which suggests an association between high cumulative dose of melphalan and eye damage [20]. Additionally, Suzuki et al. proposed that IAC seems to be less effective for treating subretinal or vitreous seeds which is due to poor penetration of the drug into the subretinal space or vitreous cavity [20]. This is in keeping with the findings published by Ghassemi et al. [31], Akyüz et al. [34], and Ong et al. [35]. However, a combination of IAC and intravitreal chemotherapy might be able to address this problem as suggested by Shields et al. [21].

Muen et al. concluded that the intraophthalmic artery melphalan allows to achieve a high level of tumour control [28]. They admitted though that the risk of severe ocular adverse events has to be considered before starting the treatment [28].

Venturi et al. mentioned that patients undergoing IAC treatment were exposed to the fluoroscopic radiation during image acquisition [30]. It is believed that this exposure is minimal, and the dose received by the brain, thorax, abdomen, and pelvis is considered to be safe [30]. Nevertheless, fluoroscopy should be used with caution taking into consideration that the children with germline mutation are irradiation sensitive which puts them at a greater risk for developing radiation-related neoplasms [30].

Shields et al. reported their 5-year experience with IAC in retinoblastoma and found that it is effective for both treatment-naive and previously treated eyes [21].

Abramson et al. emphasized that IAC allowed to achieve a significantly higher overall globe salvage rate for group D retinoblastoma than systemic chemotherapy, 79% compared to 10–47%, respectively [22]. Additionally, Abramson et al. commented on the success rate noted in the treatment naive group versus the pretreated group [22]. Their finding was that the globe salvage rate for the previously treated eyes is lower than for treatment naive eyes [22]. This might be due to the possibility that the eyes which failed previous treatment were most difficult to cure from the beginning or simply the eyes just tend to achieve better outcomes if treated with IAC from the outset [22]. Therefore, an important question emerges whether IAC should be widely used as a primary treatment rather than mainly as salvage therapy following failure of other treatments. In order to answer this question, more studies providing high-level evidence are needed.

Munier et al. in their study compared intraarterial with intravenous chemotherapy as a first-line treatment in unilateral group D retinoblastoma [23]. They found that eyes that received primary treatment with IAC have a shorter period of therapy, higher ocular survival rate, and better visual acuity compared to the group receiving systemic chemotherapy [23]. Importantly, Munier et al. commented on the fact that IAC in advanced retinoblastoma might be associated with a higher risk of metastases [23]. This is based on the fact that increased histopathological risk factors were found in group D eyes that were initially treated with enucleation [23]. The future might bring a solution to this problem. Introducing high-resolution MRI might help in detecting radiological risk factors for disseminated disease (such as choroidal invasion) and therefore establishing which eyes require adjuvant systemic chemotherapy [23]. Additionally, micrometastases might become detectable with the use of cone-rod homeobox mRNA combined with the molecular detection of GD2 synthase mRNA in the cerebrospinal fluid as described by Torbidoni et al. [57].

Francis et al. in their study reported some interesting findings not mentioned in the previous studies. They found that recurrent disease might be developed in around 25% of eyes initially treated with OAC, and the majority of recurrences happen within the first year following completion of the treatment [24]. This proves that close monitoring in this time frame is essential in order to detect the recurrent disease as soon as possible [24]. Additionally, the time interval between OAC sessions of more than 4 weeks apart and administration of the drug via nonophthalmic artery routes were both associated with a higher rate of recurrence [24].

According to Hua et al., IAC is an effective second-line treatment for group D retinoblastoma [25]. However, it should be used with caution in group E tumours [25].

## 7. Conclusion

In summary, the authors agree that intraarterial chemotherapy is an effective treatment for advanced retinoblastoma, especially group D. It allows to save the globe and does not compromise patients' survival. Local and systemic complications appear to be acceptable. Nonetheless, risks and benefits should be assessed individually in each case. The role of IAC in less advanced tumours is yet to be established. Ophthalmic artery chemosurgery should be used with caution in group E retinoblastoma as the previous studies reported that the globe salvage rate in this group is lower compared to group D eyes and there is a higher risk of developing metastatic disease.

Future work should focus on conducting larger prospective studies with a longer follow-up period to provide clinicians with high-level evidence that can be used to guide their management decisions. Moreover, it will be beneficial to explore the potential role of intraarterial chemotherapy in the treatment of less-advanced tumours.

Additionally, various novel therapies for the management of retinoblastoma are currently being explored and their results seem to be promising. Clinicians shall await the results of ongoing and future clinical trials.

## Figures and Tables

**Table 1:** International Classification of Intraocular Retinoblastoma [1].

Group	Description	Specific features
A	Very low risk: Small tumours located away from the foveola or optic disc	Tumour ≤3 mm in basal dimension or thickness

B	Low risk: Retinal tumours of any size or location not in group A without vitreous or subretinal seeding	Tumour >3 mm in basal dimension or thickness, or any of the following: (i) Macular location ≤3 mm to foveola (ii) Juxtapapillary location ≤1.5 mm to disc (iii) Clear subretinal fluid ≤3 mm from margin

C	Moderate risk: Retinal tumours of any size and location with focal vitreous or subretinal seeding	One of the following: (i) Subretinal seeds ≤3 mm from tumour (ii) Vitreous seeds ≤3 mm from tumour (iii) Both subretinal and vitreous seeds ≤3 mm from tumour (iv) Less than one quadrant of subretinal fluid in the fundus

D	High risk: Massive tumours and/or diffuse vitreous or subretinal seeding	One of the following: (i) Subretinal seeds >3 mm from tumour (ii) Vitreous seeds >3 mm from tumour (iii) Both subretinal and vitreous seeds >3 mm from tumour (iv) Greater than one quadrant of subretinal fluid in the fundus

E	Very high risk: Tumour has destroyed the eye anatomically or functionally	Extensive retinoblastoma or one of the following: (i) Irreversible neovascular glaucoma (ii) Massive intraocular haemorrhage (iii) Invasion of postlaminar optic nerve, choroid (>2 mm), sclera, orbit, anterior chamber (iv) Tumour anterior to the anterior vitreous face (v) Diffuse infiltrating tumour (vi) Phthisis bulbi or prephthisis (vii) Aseptic orbital cellulitis

**Table 2:** Reese–Ellsworth classification for retinoblastoma [14].

Group	Likelihood of globe salvage	Specific features (DD: disc diameter)
I	Very favourable	Solitary tumour <4 DD in size, at or behind the equator Multiple tumours, none >4 DD in size, all at or behind the equator

II	Favourable	Solitary tumour 4–10 DD in size, at or behind the equator Multiple tumours 4–10 DD in size, behind the equator

III	Doubtful	Any lesion anterior to the equator Solitary tumour >10 DD behind the equator

IV	Unfavourable	Mulitple tumours, some >10 DD Any lesion extending anteriorly to the ora serrata

V	Very unfavourable	Massive tumour involving over half the retina Vitreous seeding

**Table 3:** Investigations performed in retinoblastoma and their findings [1, 11, 15].

Investigation	Findings/comments
Red reflex testing	Absence of red reflex

Examination under anaesthesia (ophthalmoscopy)	White, friable retinal mass, tumour calcification, microvasculature, seeding

B-scan ocular ultrasound	Mass with high internal reflectivity due to calcifications

Fundus fluorescein angiography (FFA)	Dilated feeder vessels in the arterial phase, patchy hyperfluorescence in the venous phase, late staining

MRI of the brain and orbits	Hyperintense lesion on T1, hypointense lesion on T2, evaluation of tumour size, optic nerve involvement, detection of extraocular extension and pinealoblastoma, does not detect calcifications

CT of the brain and orbits	Hyperdense lesion compared to surrounding vitreous, possible calcifications, usually avoided due to increased risk of secondary cancers

**Table 4:** Summary of IAC chemotherapeutic agents [5].

Name of drug	Type of drug	Standard dose and dose range in mg	Indications
Melphalan	Alkylating agent, nitrogen mustard derivative	5 (3–7.5)	Group B and C tumours as a single agent

Topotecan	Camptothecin derivative, topoisomerase 1 inhibitor	1 (1-2)	Advanced retinoblastoma with diffuse vitreous seeds

Carboplatin	Platinum-based derivative	20 (15–30)	Bilateral IAC to lower the cumulative toxicity of melphalan, recurrence after IAC, suboptimal response to combined melphalan and topotecan

**Table 5:** Overall globe salvage and globe salvage by groups across analysed studies.

Study	Overall globe salvage (%)	Globe salvage by groups
Gobin et al. [7]	80	RE I–IV 100%, RE V 77%, 81.7% primary, 58.4% secondary
Peterson et al. [26]	76	76% D
Suzuki et al. [20]	60	100% A, 88% B, 65% C, 45% D, 30% E
Marr et al. [27]	88	No data
Muen et al. [28]	80	80% secondary
Thampi et al. [29]	70	86% A–C, 38% D and E, 58.3% primary, 87.5% secondary
Venturi et al. [30]	79	57% primary, 95.5% secondary
Ghassemi et al. [31]	62.5	67.5% D-E, 84% primary, 56% secondary
Shields et al. [21]	67	100% B, 100% C, 94% D, 36% E of primary treatments, 72% primary, 62% secondary
Parareda et al. [32]	58	58% D
Akyüz et al. [33]	66	75% primary, 64% secondary
Ong et al. [34]	59	75% B and C, 54% D and E, 67% primary, 55% secondary
Abramson et al. [22]	78.6	78.6% D, 85% primary, 74% secondary
Michaels et al. [35]	58	43% primary, 67% secondary
Tuncer et al. [36]	66.6	66.6% D, 66.6% primary
Chen et al. [37]	78.5	100% B, 100% C, 78.6% D, 62% E, 93.3% primary, 79% secondary
Munier et al. [23]	100	100% D
Reddy et al. [38]	66	66% secondary
Francis et al. [24]	92	No data
Funes et al. [39]	63	92% B-C, 40% D, 69% primary, 68% secondary
Hua et al. [25]	30	42% D, 21% E
Wang et al. [40]	78.7	84.2% D, 69.6% E, 78.7% primary
Dalvin et al. [41]	74	B 100%, C 100%, D 79%, E 58%

**Table 6:** Incidence of metastases, deaths, and secondary tumours reported in the analysed studies.

Study	Metastasis (no of pts)	Deaths (no of pts)	Secondary tumour (no of tumours)
Gobin et al. [7]	2	0	0
Suzuki et al. [20]	8	12	12
Akyüz et al. [33]	2	2	0
Ong et al. [34]	3	2	0
Abramson et al. [22]	3	1	0
Francis et al. [24]	5	6	7
Funes et al. [39]	0	2	2

**Table 7:** Sample size, technical success rate, and duration of follow-up across analysed studies.

Study	No. of eyes	Technical success rate of catheterization (%)	Follow-up in months (range)
Gobin et al. [7]	95	98.5	Median 13 (1–29)
Peterson et al. [26]	17	100	Mean 8.6 (3–12)
Suzuki et al. [20]	408	98.8	Median 74 (0–252)
Marr et al. [27]	26	100	Mean 14 (1–43)
Muen et al. [28]	15	No data	Mean 9 (3–16)
Thampi et al. [29]	20	100	Median 14.5 (1–29)
Venturi et al. [30]	39	94.7	Mean 13 (1–27)
Ghassemi et al. [31]	24	No data	Mean 17.6 (3–57)
Shields et al. [21]	70	99.5	Mean 19
Parareda et al. [32]	12	94	Median 30 (6–57)
Akyüz et al. [33]	56	No data	Median 12 (0–28)
Ong et al. [34]	17	91	Median 22 (5–43)
Abramson et al. [22]	112	No data	Mean 34 (2–110)
Michaels et al. [35]	19	100	Median 13
Tuncer et al. [36]	24	97.3	Median 29 (6–55)
Chen et al. [37]	107	98.5	Mean 14 (3–28)
Munier et al. [23]	25	No data	Mean 42 (20–90)
Reddy et al. [38]	9	No data	Median 21 (4–35)
Francis et al. [24]	436	No data	Median 27 (0–120)
Funes et al. [39]	97	99	Median 49 (12–79)
Hua et al. [25]	84	94.5	Mean 14 (3–28)
Wang et al. [40]	61	100	No data
Dalvin et al. [41]	54	No data	27 (21–63)

## Supplementary Materials

Supplementary Materials Supplementary material 1 contains a table summarising baseline characteristics of the included studies. Supplementary material 2 contains a table summarising treatment outcomes, complications, and follow-up period of the included studies.

## References

[1] Kaufman P. L., Kim J., Berry J. L. (2018). Retinoblastoma: clinical presentation, evaluation, and diagnosis. https://www-uptodate-com.libproxy.ucl.ac.uk/contents/retinoblastoma-clinical-presentation-evaluation-and-diagnosis?search=retinoblastoma&source=search_result&selectedTitle=1~79&usage_type=default&display_rank=1.

[2] Rao R., Honavar S. G. (2017). Retinoblastoma. *The Indian Journal of Pediatrics*.

[3] Pandey A. N. (2014). Retinoblastoma: an overview. *Saudi Journal of Ophthalmology*.

[4] Naseripour M. (2012). Retinoblastoma survival disparity: the expanding horizon in developing countries. *Saudi Journal of Ophthalmology*.

[5] Manjandavida F. P., Stathopoulos C., Zhang J., Honavar S. G., Shields C. L. (2019). Intra-arterial chemotherapy in retinoblastoma—a paradigm change. *Indian Journal of Ophthalmology*.

[6] Yamane T., Kaneko A., Mohri M. (2004). The technique of ophthalmic arterial infusion therapy for patients with intraocular retinoblastoma. *International Journal of Clinical Oncology*.

[7] Gobin Y. P., Dunkel I. J., Marr B. P. (2011). Intra-arterial chemotherapy for the management of retinoblastoma: four-year experience. *Archives of Ophthalmology*.

[8] Nichols K. E., Walther S., Chao E., Shields C., Ganguly A. (2009). Recent advances in retinoblastoma genetic research. *Current Opinion in Ophthalmology*.

[9] Bestetti I., Sironi A., Catusi I. (2018). 13q mosaic deletion including RB1 associated to mild phenotype and no cancer outcome—case report and review of the literature. *Molecular Cytogenetics*.

[10] Almater A., Alfaleh A., Alshomar K., AlMesfer S., Alkatan H. (2019). Retinoblastoma: update on current management. *Retinoblastoma—Past, Present and Future*.

[11] Kanski J., Bowling B. (2016). *Kanski’s Clinical Ophthalmology*.

[12] Rushlow D. E., Mol B. M., Kennett J. Y. (2013). Characterisation of retinoblastomas without RB1 mutations: genomic, gene expression, and clinical studies. *The Lancet Oncology*.

[13] Fabian I. D., Sagoo M. S., Chaugule S. S., Honavar S. G., Finger P. T. (2019). Overview of Intraocular Tumours. *Surgical Ophthalmic Oncology: A Collaborative Open Access Reference*.

[14] Chawla B., Jain A., Azad R. (2013). Conservative treatment modalities in retinoblastoma. *Indian Journal of Ophthalmology*.

[15] Albader F., Fatani D., Alkatan H. (2019). Uses of radiological imaging in retinoblastoma. *Retinoblastoma-Past, Present and Future*.

[16] Kaufman P. L., Kim J., Berry J. L. (2018). Retinoblastoma: Treatment and outcome. https://www-uptodate-com.libproxy.ucl.ac.uk/contents/retinoblastoma-treatment-and-outcome?search=retinoblastoma&source=search_result&selectedTitle=2%7E79&usage_type=default&display_rank=2.

[17] Inomata M., Kaneko A. (1987). Chemosensitivity profiles of primary and cultured human retinoblastoma cells in a human tumor clonogenic assay. *Japanese Journal of Cancer Research*.

[18] Zanaty M., Barros G., Chalouhi N. (2014). Update on intra-arterial chemotherapy for retinoblastoma. *The Scientific World Journal*.

[19] Guasti A., Leonini S., Bertelli E. (2019). Intra-arterial chemotherapy for retinoblastoma: the dosimetric impact. *Neuroradiology*.

[20] Suzuki S., Yamane T., Mohri M., Kaneko A. (2011). Selective ophthalmic arterial injection therapy for intraocular retinoblastoma: the long-term prognosis. *Ophthalmology*.

[21] Shields C. L., Manjandavida F. P., Lally S. E. (2014). Intra-arterial chemotherapy for retinoblastoma in 70 eyes: outcomes based on the international classification of retinoblastoma. *Ophthalmology*.

[22] Abramson D. H., Daniels A. B., Marr B. P. (2016). Intra-arterial chemotherapy (ophthalmic artery chemosurgery) for group D retinoblastoma. *PLoS One*.

[23] Munier F. L., Mosimann P., Puccinelli F. (2017). First-line intra-arterial versus intravenous chemotherapy in unilateral sporadic group D retinoblastoma: evidence of better visual outcomes, ocular survival and shorter time to success with intra-arterial delivery from retrospective review of 20 years of treatment. *British Journal of Ophthalmology*.

[24] Francis J. H., Levin A. M., Zabor E. C., Pierre Gobin Y., Abramson D. H. (2018). Ten-year experience with ophthalmic artery chemosurgery: ocular and recurrence-free survival. *PLoS One*.

[25] Hua J., Hua J., Gang S., Yizhou J. (2018). Intra-arterial chemotherapy as second-line treatment for advanced retinoblastoma: a 2-year single-center study in China. *Journal of Cancer Research & Therapeutics*.

[26] Peterson E. C., Elhammady M. S., Quintero-Wolfe S., Murray T. G., Aziz-Sultan M. A. (2011). Selective ophthalmic artery infusion of chemotherapy for advanced intraocular retinoblastoma: initial experience with 17 tumors. *Journal of Neurosurgery*.

[27] Marr B. P., Brodie S. E., Dunkel I. J., Gobin Y. P., Abramson D. H. (2012). Three-drug intra-arterial chemotherapy using simultaneous carboplatin, topotecan and melphalan for intraocular retinoblastoma: preliminary results. *British Journal of Ophthalmology*.

[28] Muen W. J., Kingston J. E., Robertson F., Brew S., Sagoo M. S., Reddy M. A. (2012). Efficacy and complications of super-selective intra-ophthalmic artery melphalan for the treatment of refractory retinoblastoma. *Ophthalmology*.

[29] Thampi S., Matthay, Hetts (2013). Superselective intra-arterial melphalan therapy for newly diagnosed and refractory retinoblastoma: results from a single institution. *Clinical Ophthalmology*.

[30] Venturi C., Bracco S., Cerase A. (2013). Superselective ophthalmic artery infusion of melphalan for intraocular retinoblastoma: preliminary results from 140 treatments. *Acta Ophthalmologica*.

[31] Ghassemi F., Ghanaati H., Karkhaneh R., Boujabadi L., ZiaTabatabaie S., Taher Rajabi M. (2014). Outcome of retinoblastoma following limited sessions of intra-arterial chemotherapy in Iran. *Iranian Journal of Radiology*.

[32] Parareda A., Català J., Carcaboso A. M. (2014). Intra-arterial chemotherapy for retinoblastoma. Challenges of a prospective study. *Acta Ophthalmologica*.

[33] Akyüz C., Kıratlı H., Şen H., Aydın B., Tarlan B., Varan A (2015). Intra-arterial chemotherapy for retinoblastoma: a single-center experience. *Ophthalmologica*.

[34] Ong S. J., Chao A.-N., Wong H.-F., Liou K.-L., Kao L.-Y. (2015). Selective ophthalmic arterial injection of melphalan for intraocular retinoblastoma: a 4-year review. *Japanese Journal of Ophthalmology*.

[35] Michaels S. T., Abruzzo T. A., Augsburger J. J., Corrêa Z. M., Lane A., Geller J. I. (2016). Selective ophthalmic artery infusion chemotherapy for advanced intraocular retinoblastoma: CCHMC early experience. *Journal of Pediatric Hematology/Oncology*.

[36] Tuncer S., Sencer S., Kebudi R., Tanyıldız B., Cebeci Z., Aydın K. (2016). Superselective intra-arterial chemotherapy in the primary management of advanced intra-ocular retinoblastoma: first 4-year experience from a single institution in Turkey. *Acta Ophthalmologica*.

[37] Chen M., Jiang H., Zhang J. (2017). Outcome of intra-arterial chemotherapy for retinoblastoma and its influencing factors: a retrospective study. *Acta Ophthalmologica*.

[38] Reddy M. A., Naeem Z., Duncan C. (2017). Reduction of severe visual loss and complications following intra-arterial chemotherapy (IAC) for refractory retinoblastoma. *British Journal of Ophthalmology*.

[39] Funes S., Sampor C., Villasante F. (2018). Feasibility and results of an intraarterial chemotherapy program for the conservative treatment of retinoblastoma in Argentina. *Pediatric Blood & Cancer*.

[40] Wang L., Han M., Zhao J. (2018). Intra-arterial chemotherapy for unilateral advanced intraocular retinoblastoma: results and short-term complications. *Medicine*.

[41] Dalvin L. A., Kumari M., Essuman V. A. (2019). Primary intra-arterial chemotherapy for retinoblastoma in the intravitreal chemotherapy era: five years of experience. *Ocular Oncology and Pathology*.

[42] Houston S. K., Lampidis T. J., Murray T. G. (2013). Models and discovery strategies for new therapies of retinoblastoma. *Expert Opinion on Drug Discovery*.

[43] Turhan S., Cekic O. (2014). New hopes in retinoblastoma treatment. *International Journal of Hematology and Oncology*.

[44] Goldsmith Z. K., Coppess W., Irvine A. S. (2018). Targeting the platelet-derived growth factor-beta stimulatory circuitry to control retinoblastoma seeds. *Investigative Opthalmology & Visual Science*.

[45] Brennan R. C., Federico S., Bradley C. (2011). Targeting the p53 pathway in retinoblastoma with subconjunctival nutlin-3a. *Cancer Research*.

[46] Boutrid H., Jockovich M.-E., Murray T. G. (2008). Targeting hypoxia, a novel treatment for advanced retinoblastoma. *Investigative Opthalmology & Visual Science*.

[47] Piña Y., Houston S. K., Murray T. G. (2010). Focal, periocular delivery of 2-deoxy-d-glucose as adjuvant to chemotherapy for treatment of advanced retinoblastoma. *Investigative Opthalmology & Visual Science*.

[48] Zhang D., Li J., Wang F., Hu J., Wang S., Sun Y. (2014). 2-Deoxy-D-glucose targeting of glucose metabolism in cancer cells as a potential therapy. *Cancer Letters*.

[49] Pina Y., Decatur C., Murray T. (2011). Advanced retinoblastoma treatment: targeting hypoxia by inhibition of the mammalian target of rapamycin (mTOR) in LH<inf>BETA</inf>T<inf>AG</inf> retinal tumors. *Clinical Ophthalmology*.

[50] Dalgard C. L., Van Quill K. R., O’Brien J. M. (2008). Evaluation of the *in vitro* and *in vivo* antitumor activity of histone deacetylase inhibitors for the therapy of retinoblastoma. *Clinical Cancer Research*.

[51] Theodoropoulou S., Kolovou P. E., Morizane Y. (2010). Retinoblastoma cells are inhibited by aminoimidazole carboxamide ribonucleotide (AICAR) partially through activation of AMP-dependent kinase. *The FASEB Journal*.

[52] Jo D. H., Kim J. H., Kim J. H. (2019). Targeting tyrosine kinases for treatment of ocular tumors. *Archives of Pharmacal Research*.

[53] Zhan W.-J., Zhu J.-F., Wang L.-M. (2016). Inhibition of proliferation and induction of apoptosis in RB116 retinoblastoma cells by afatinib treatment. *Tumor Biology*.

[54] Shao Y., Yu Y., Zong R. (2017). Erlotinib has tumor inhibitory effect in human retinoblastoma cells. *Biomedicine & Pharmacotherapy*.

[55] De Moura L. R., Marshall J.-C., Di Cesare S., Fernandes B. F., Antecka E., Burnier M. N. (2013). The effect of imatinib mesylate on the proliferation, invasive ability, and radiosensitivity of retinoblastoma cell lines. *Eye*.

[56] Suzuki S., Kaneko A. (2004). Management of intraocular retinoblastoma and ocular prognosis. *International Journal of Clinical Oncology*.

[57] Torbidoni A. V., Laurent V. E., Sampor C. (2015). Association of cone-rod homeobox transcription factor messenger RNA with pediatric metastatic retinoblastoma. *JAMA Ophthalmology*.

