# Supplementary material for: The Role of Intraarterial Chemotherapy in the Management of Retinoblastoma
Source: J Ophthalmol. 2020 Jan 22;2020:3638410. doi: 10.1155/2020/3638410 (PMC7001664; doi:10.1155/2020/3638410)
Supplement: Supplementary Materials — Supplementary material 1 contains a table summarising baseline characteristics of the included studies. Supplementary material 2 contains a table summarising treatment outcomes, complications, and follow-up period of the included studies. [file 3638410.f1.docx]

| Study & design | Years of study, country | No of eyes | Unilateral vs bilateral disease | Eye classification (no of eyes) | Primary vs secondary tx (% of eyes) | No of IAC sessions (per eye) | Chemotherapy technique & agents | Concurrent therapies (% of eyes) | Technical  success |
| --- | --- | --- | --- | --- | --- | --- | --- | --- | --- |
| Gobin et al.,  2011 [22], prospective | 2006-2010, USA | 95 | 38% vs 62% | Reese-Ellsworth  I-III (8)  IV (4)  Va (10)  Vb (73) | 41% vs 59% | Median 3 | Superselective  Melphalan+/-  Topotecan+/-  Carboplatin+/-  Methotrexate | Yes, 77% | 98.5% |
| Peterson et al., 2011 [35], prospective | 2008-2009,  USA | 17 | 47% vs 53% | D (17) | 6% vs 94% | Mean 1.5 | Superselective  Melphalan | Not reported | 100% |
| Suzuki et al., 2011 [24], retrospective | 1988-2007,  Japan | 408 | 39% vs 61% | A (5)  B (130)  C (30)  D (216)  E (18) | 12% vs 88% | Median 3 | Balloon catheter  Melphalan | Yes, 93% | 98.8% |
| Marr et al., 2012 [36], retrospective | 2006-2011,  USA | 26 | 36% vs 64% | B (1)  C (2)  D (17)  E (6) | 32% vs 68% | Median 2 | Superselective  Ballon catheter  Melphalan+  Topotecan+  Carboplatin | Yes, 80% | 100% |
| Muen et al., 2012 [25], prospective | 2009-2010,  UK | 15 | No data | No data | 100% secondary | Median 2 | Superselective  Melphalan | Not reported | No data |
| Thampi et al., 2013 [37], retrospective | 2010-2012, USA | 20 | 38% vs 62% | A (1)  B (4)  C (2)  D (11)  E (2) | 60% vs 40% | Median 2.5 | Superselective  Melphalan | Yes, 65% | 100% |

**SUPPLEMENTARY MATERIAL 1: BASELINE CHARACTERISTICS OF THE INCLUDED STUDIES**

| Study & design | | Years of study, country | | No of eyes | Unilateral vs bilateral disease | | Eye classification (no of eyes) | | Primary vs secondary tx (% of eyes) | | No of IAC sessions (per eye) | | Chemotherapy technique & agents | | Concurrent therapies (% of eyes) | Technical  success |
| --- | --- | --- | --- | --- | --- | --- | --- | --- | --- | --- | --- | --- | --- | --- | --- | --- |
| Venturi et al.,  2013 [26],  retrospective | | 2008-2010, Italy | | 39 | Not reported | | Reese-Ellsworth  (specific data not reported) | | 41% vs 59% | | Range 1-6 | | Superselective  Melphalan | | Yes, 74% | 94.7% |
| Ghassemi et al., 2014 [27], retrospective | | 2009-2012,  Iran | | 24 | 58% vs 42% | | B (1)  C (2)  D (18)  E (3) | | 25% vs 75% | | Mean 1.4 | | Superselective  Melphalan +/-  Topotecan +/-  Carboplatin | | Yes, 67% | No data |
| Shields et al.,  2014 [28], retrospective | | 2009-2013, USA | | 70 | 63% vs 37% | | B (1)  C (4)  D (17)  E (14) of 36 primary tx | | 51% vs 49% | | Median 3 | | Superselective  Melphalan +/- Topotecan +/-  Carboplatin | | Not reported | 99.5% |
| Parareda et al., 2014 [38], prospective | | 2008-2012,  Spain | | 12 | 64% vs 36% | | D (12) | | 67% vs 33% | | Median 2.5 | | Superselective  Melphalan | | Yes, 58% | 94% |
| Akyuz et al., 2015 [29], retrospective | | 2011-2014,  Turkey | | 56 | 37% vs 63% | | A (7)  B (6)  C (16)  D (19)  E (8) | | 21% vs 79% | | Mean 2.3 | | Superselective  Melphalan | | Yes, 9% | No data |
| Ong et al., 2015 [39], retrospective | | 2010-2013, Taiwan | | 17 | 42% vs 58% | | B (3)  C (1)  D (1)  E (12) | | 35% vs 65% | | Median 3 | | Superselective  Melphalan | | Yes, 65% | 91% |
| Study & design | | Years of study, country | | No of eyes | Unilateral vs bilateral disease | | Eye classification (no of eyes) | | Primary vs secondary tx (% of eyes) | | No of IAC sessions (per eye) | | Chemotherapy technique & agents | | Concurrent therapies (% of eyes) | Technical  success |
| Abramson et al., 2016 [30], retrospective | | 2006-2012, USA | | 112 | 39% vs 61% | | D (112) | | 42% vs 58% | | Median 3 | | Superselective  Melphalan +/- Topotecan +/-  Carboplatin | | Yes, all patients | No data |
| Michaels et al., 2016 [40], retrospective | | 2008-2013,  USA | | 19 | 88% vs 12% | | C (3)  D (15)  E (1) | | 41% vs 59% | | Mean 5 | | Superselective  Melphalan +/- Topotecan +/-  Carboplatin | | Yes, 65% | 100% |
| Tuncer et al., 2016 [41], prospective | | 2011-2015,  Turkey | | 24 | 91% vs 9% | | D (24) | | 100% primary | | Mean 3 | | Superselective  Melphalan +/- Topotecan +/-  Carboplatin | | Yes, 33% | 97.3% |
| Chen et al., 2017 [42], retrospective | | 2011-2013,  China | | 107 | 33% vs 67% | | B (11)  C (11)  D (56)  E (29) | | 28% vs 72% | | Mean 3 | | Superselective  Melphalan+/-  Topotecan | | Not reported | 98.5% |
| Munier et al., 2017 [31], retrospective, comparative | | 1997-2014,  Switzerland | | 25 | 100% unilateral | | D (25) | | 100% primary | | Mean 2.7 | | Superselective  Melphalan | | Yes, 80% | No data |
| Reddy et al., 2017 [43],  retrospective | | 2013-2015,  UK | | 9 | 44% vs 56% | | A (2)  B (2)  C (2)  D (3) | | 100% secondary | | Median 3 | | Superselective  Melphalan+/-  Topotecan | | Yes, 44% | No data |
| Francis et al., 2018 [32], retrospective | | 2006-2017,  USA | | 436 | 38% vs 62% | | A (3)  B (42)  C (45)  D (208)  E (85) NA (53) | | 52% vs 48% | | Median 3 | | Superselective  Balloon catheter  Melphalan+/-  Topotecan+/-  Carboplatin | | Yes | No data |
| Study & design | Years of study, country | | No of eyes | | | Unilateral vs bilateral disease | | Eye classification (no of eyes) | | Primary vs secondary tx (% of eyes) | | No of IAC sessions (per eye) | | Chemotherapy technique & agents | Concurrent therapies (% of eyes) | Technical  success |
| Funes et al., 2018 [33], retrospective | 2010-2015,  Argentina | | 97 | | | 25% vs 75% | | No data | | 36% vs 64% | | Median 4 | | Superselective  Melphalan+/-  Topotecan+/-  Carboplatin | Yes | 99% |
| Hua et al., 2018 [34], retrospective | 2013-2015,  China | | 84 | | | 65% vs 35% | | D (36)  E (48) | | 100% secondary | | Mean 2.8 | | Superselective  Melphalan+  Topotecan | Yes | 94.5% |
| Wang et al., 2018 [44], retrospective | 2016-2017,  China | | 61 | | | 100% unilateral | | D (38)  E (23) | | 100% primary | | Median 3.1 | | Superselective  Melphalan +/- Topotecan +/-  Carboplatin | Yes | 100% |
| Dalvin et al., 2019 [45], retrospective | 2012-2017,  USA | | 54 inc.  *34 IAC  *20 IAC+IViC | | | 98% vs 2% | | B (2)  C (3)  D (30)  E (19) | | 100% primary | | Mean 3.4 | | Superselective  Melphalan +/-  Topotecan | Yes, 37% | No data |

| Study | Overall globe salvage | | Globe salvage by groups | | Mets  (no of cases) | | Death  (no of cases) | | Ocular complications  (no of cases) | | Extraocular complications  (no of cases) | | Vitreous/ subretinal seeding | | 2ry cancer  (no of cases) | | Follow up  (months) | |  |
| --- | --- | --- | --- | --- | --- | --- | --- | --- | --- | --- | --- | --- | --- | --- | --- | --- | --- | --- | --- |
| Gobin et al., 2011 [22] | 80% | | RE I-IV 100%  RE V 77%  *81.7% primary*  *58.4% secondary* | | 2 | | 0 | | Periocular oedema and redness (10)  Avascular retinopathy (4)  Loss of eyelashes (12) | | Superficial femoral artery occlusion (1)  Bronchospasm (24)  Allergic reaction to iodinated contrast (6)  Forehead hyperaemia (14)  Neutropenia (29) Neutropenic fever (1) | | No data | | 0 | | Median 13 (1-29) | |  |
| Peterson et al., 2011 [35] | 76% | | 76% D | | 0 | | 0 | | Delayed vitreous haemorrhage (4)  Retinal ischaemia (1) | | Neutropenia (2)  **SUPPLEMENTARY MATERIAL 2: TREATMENT OUTCOME, COMPLICATIONS & FOLLOW-UP**  Fever (1)  Groin hematoma (1) | | No data | | 0 | | Mean 8.6 months (3-12) | |  |
| Suzuki et al., 2011 [24] | 60% | | 100% A  88% B  65% C  45% D  30% E | | 8 | | 12 | | Orbital inflammation (2)  Chorioretinal atrophy (2)  Periocular swelling and erythema (some cases)  Retinal haemorrhage | | Bronchospasm (1)  Bradycardia (24)  Vomiting (58) | | Mostly no regression of subretinal/  vitreous seeds | | 12 ( all pts received EBRT) | | Median 74  (0-252) | |  |
| Marr et al., 2012 [36] | 88% | | No data | | 0 | | 0 | | Not reported | | Neutropenia (16) | | No data | | 0 | | Mean 14 (1-43) | |  |
| Muen et al., 2012 [25] | 80% | | *80% secondary* | | 0 | | 0 | | III CN palsy (6)  Orbital oedema (3)  Permanent RD (1)  Vitreous haemorrhage (4)  RPE changes (7) | | Forehead erythema (3)  Anaphylactoid reaction post contrast injection (1) | | No data | | 0 | | Mean 9 months (3-16) | |  |
| Study | | Overall globe salvage | | Globe salvage by groups | | Mets  (no of cases) | | Death  (no of cases) | | Ocular complications  (no of cases) | | Extraocular complications  (no of cases) | | Vitreous/ subretinal seeding | | 2ry cancer  (no of cases) | | Follow up  (months) | |
| Thampi et al., 2013 [37] | | 70% | | 86% A-C  38% D and E  *58.3% primary*  *87.5% secondary* | | 0 | | 0 | | vitreous haemorrhage (3) eyelid oedema and erythema (2)  dacryohemorrhea (1)  cataract (1)  chorioretinal atrophy (1)  ophthalmic artery spasm (1) | | Postop fever (4)  neutropenia (2)  bronchospasm (1) | | Regression of local vitreous seeds in all patients | | 0 | | Median 14.5  (1-29) | |
| Venturi et al.,2013 [26] | | 79% | | *57% primary*  *95.5% secondary* | | 0 | | 0 | | Chorioretinal atrophy (2)  Ptosis (10)  Strabismus/ exotropia (1)  Eyelid hyperaemia and oedema (25)  Loss of lashes (2) | | Bronchospasm  Bradycardia and hypotension  Neutropenia (2)  Frontal alopecia (2)  Frontal rash (10) | | No data | | 0 | | Mean 13  (1-27) | |
| Ghassemi et al., 2014 [27] | | 62.5% | | 67.5% D-E  *84% primary*  *56% secondary* | | 0 | | 0 | | Eyelid oedema (12)  Vitreous haemorrhage (9)  Preretinal haemorrhage (3)  Subretinal heamorhage (1)  CRAO (2)  Retinal detachment (3)  Chorioretinal atrophy (3)  NV glaucoma (1)  Phthisis bulbi (2)  Ptosis (2)  Iris atrophy (1)  Arteriolitis (1) | | Neutropenia | | Complete regression of vitreous seeds in 25% of eyes, partial regression in 37.5% of eyes | | 0 | | Mean 17.6  (3-57) | |

| Study | Overall globe salvage | Globe salvage by groups | Mets  (no of cases) | Death  (no of cases) | Ocular complications  (no of cases) | Extraocular complications  (no of cases) | Vitreous/ subretinal seeding | 2ry cancer  (no of cases) | Follow up  (months) |
| --- | --- | --- | --- | --- | --- | --- | --- | --- | --- |
| Shields et al., 2014 [28] | 67% | 100% B  100% C  94% D  36% E of primary treatments  *72% primary*  *62% secondary* | 0 | 0 | Eyelid oedema (10)  Blepharoptosis (10)  Vitreous haemorrhage (4)  BRAO (2)  Ophthalmic artery spasm (4)  Ophthalmic artery occlusion (3)  Partial choroidal ischaemia (4)  Optic neuropathy (1)  Phthisis bulbi (1) | Forehead hyperaemia (3)  Scalp alopecia (1) | Complete regression of vitreous seeds in 87% and subretinal seeds in 95% | 0 | Mean 19 |
| Parareda et al., 2014 [38] | 58% | 58% D | 0 | 0 | Arteriolar sclerosis (2)  RPE hyperpigmentation (2)  Partial retinal atrophy (2)  Eyelid oedema (3)  Ptosis (1)  Choroidal occlusion (4)  OA spasm (1) | None | No data | 0 | Median 30  (6-57) |
| Akyuz et al., 2015 [29] | 66% | *75% primary*  *64% secondary* | 2 (brain) | 2 | Eyelid oedema (22)  Conjunctival chemosis (12)  Ptosis (5)  Frontal erythema (3)  Limited eye movements (3)  Proptosis (1)  RPE changes (30)  Optic atrophy (3) | None | Improvement in vitreous seeding in 33% of eyes | 0 | Median 12 (0-28) |

| Study | | | Overall globe salvage | | | Globe salvage by groups | Mets  (no of cases) | | | | Death  (no of cases) | | | | Ocular complications  (no of cases) | | | Extraocular complications  (no of cases) | Vitreous/ subretinal seeding | | 2ry cancer  (no of cases) | | Follow up  (months) |
| --- | --- | --- | --- | --- | --- | --- | --- | --- | --- | --- | --- | --- | --- | --- | --- | --- | --- | --- | --- | --- | --- | --- | --- |
| Ong et al., 2015 [39] | | | 59% | | | 75% B and C  54% D and E  *67% primary*  *55% secondary* | 3 | | | | 2 | | | | lid oedema (2)  III CN palsy (2)  VI CN palsy (1)  chorioretinal atrophy (6)  retinal arterial occlusion (3)  RD (1)  VH (7) | | | Neutropenic fever (1) | partial or no prominent regression of vitreous seeds in 71% of pts | | 0 | | Median 22  (5-43) |
| Abramson et al., 2016 [30] | | | 78.6% | | | 78.6% D  *85% primary*  *74% secondary* | 3 (all survived) | | | | 1 (pinealoblastoma) | | | | Eyelid oedema and erythema (25)  Madarosis (10)  Retinal or choroidal vascular occlusions (6)  Phthisis (5)  Vitreous haemorrhage (4)  Ptosis (4)  Optic nerve swelling (3)  Retinopathy (2)  Cranial nerve palsy (2)  Ophthalmic artery injury (2)  Suprachoroidal haemorrhage (1) | | | Neutropenia (39)  Bronchospasm (44)  Allergy type reaction (5)  Thrombocytopenia (4)  Fever (4)  Cardiorespiratory side effects (3)  Injection site complications (3)  Epistaxis (1) | No data | | 0 | | Mean 34 (2-110) |
| Study | Overall globe salvage | | | Globe salvage by groups | | | | Mets  (no of cases) | | | | Death  (no of cases) | | Ocular complications  (no of cases) | | | Extraocular complications  (no of cases) | | | Vitreous/ subretinal seeding | | 2ry cancer  (no of cases) | Follow up  (months) |
| Michaels et al., 2016 [40] | 58% | | | *43% primary*  *67% secondary* | | | | 0 | | | | 0 | | Localized erythema (14)  Eyelid oedema (12)  Eyebrow/eyelash loss (7)  Chemosis (4) | | | Forehead hyperpigmentation (5)  Grade 3 neutropenia (11)  Grade 4 neutropenia (5)  Neutropenic fever (1)  Nausea and vomiting (9)  Fever (5)  Bronchospasm (4)  Carboplatin anaphylaxis (1)  Lower extremity arterial thrombosis (1)  Cerebral vasoconstriction (1) | | | No data | | 0 | Median 13 |
| Tuncer et al., 2016 [41] | 66.6% | | | 66.6% D  *66.6% primary* | | | | 0 | | | | 0 | | Eyelid oedema (13)  Ptosis (6)  Chorioretinal atrophy (9)  Retinal detachment (5)  Vitreous haemorrhage (1)  Neovascular glaucoma (1) | | | Anaphylactoid reaction (1) Forehead hyperpigmentation (3)  Nausea and vomiting (8) | | | Complete regression of subretinal seeds in 92% of eyes, complete regression of vitreous seeds in 50% of eyes | | 0 | Median 29  (6-55) |
| Study | | Overall globe salvage | | | Globe salvage by groups | | | | Mets  (no of cases) | Death  (no of cases) | | | Ocular complications  (no of cases) | | | Extraocular complications  (no of cases) | | | Vitreous/ subretinal seeding | | 2ry cancer  (no of cases) | | Follow up  (months) |
| Chen et al., 2017 [42] | | 78.5% | | | 100% B  100% C  78.6% D  62% E  *93.3% primary*  *79% secondary* | | | | 0 | 0 | | | Eyelid oedema (15)  Conjunctival congestion (32)  Excessive tearing (10)  Vitreous haemorrhage (9)  Subretinal haemorrhage (10)  Retinal vasculopathy (8)  OA spasm with reperfusion (5) | | | Internal carotid artery spasm (5)  Fever (16)  Vomiting (20)  Transient myelosuppression (10) | | | No data | | 0 | | Mean 14  (3-28) |
| Munier et al., 2017 [31] | | 100% | | | 100% D | | | | 0 | 0 | | | Retinal detachment (14)  Retinopathy (8)  Vitreous haemorrhage (1)  Neovascularisation (2)  Cataract (6)  Eyelid oedema (5)  Eyelid erythema (1)  Ptosis (1) | | | Frontal hair loss (1)  Cardiorespiratory disturbances (4)  ICA/OA spasm (2)  Neutropenia (3)  Nausea (4) | | | Complete regression of subretinal seeds in 78% of eyes, complete regression of vitreous seeds in 80% | | 0 | | Mean 42 (20-90) |
| Reddy et al., 2017 [43] | | 66% | | | *66% secondary* | | | | 0 | 0 | | | nasal choroidal ischaemia (1)  sixth nerve palsy (1)  ptosis (2)  sluggish pupil (1) | | | Severe autonomic episode (6) | | | No data | | 0 | | Median 21 (4-35) |
| Francis et al., 2018 [32] | | 92% | | | No data | | | | 5 | 6 | | | No data | | | No data | | | No data | | 7 | | Median 27 (0-120) |
| Dalvin et al., 2019 [45] | | 74% | | | B 100%  C 100%  D 79%  E 58% | | | | 0 | 0 | | | Not specified | | | Not specified | | | No data | | 0 | | 27 (21-63) |

| Study | Overall globe salvage | Globe salvage by groups | Mets  (no of cases) | Death  (no of cases) | Ocular complications  (no of cases) | Extraocular complications  (no of cases) | Vitreous/ subretinal seeding | 2ry cancer  (no of cases) | Follow up  (months) |
| --- | --- | --- | --- | --- | --- | --- | --- | --- | --- |
| Funes et al., 2018 [33] | 63% | 92% B-C  40% D  *69% primary*  *68% secondary* | 0 | 2 | Eyelid oedema (9)  III CN palsy (2)  Retinal/choroidal vascular occlusions (3)  Ptosis (1) | Fever and neutropenia (5)  Hypotension and bradycardia (2)  Femoral thrombosis (1)  Thrombocytopenia (8)  Neutropenia (24)  Bronchospasm (3)  VZV infection (1)  Respiratory infection (1)  Frontal erythema/alopecia (12) | No data | 2  (bone Ewing sarcoma/cranio-pharyn-gioma) | Median 49 ( 12-79) |
| Hua et al., 2018, [34] | 30% | 42% D  21% E | 0 | 0 | Eyelid oedema (12)  Chemosis (25)  Excessive tearing (10)  Vitreous haemorrhage (7)  Subretinal haemorrhage (9)  Retinal vasculopathy (6)  OA spasm with reperfusion (11) | Fever (14)  Vomiting (17)  Transient myelosuppression (8) | No data | 0 | Mean 14 ( 3-28) |
| Wang et al., 2018 [44] | 78.7% | 84.2% D  69.6% E  *78.7% primary* | Not reported | Not reported | Lid oedema (15)  Ptosis (5)  Forehead congestion (3)  Retinal haemorrhage (5)  Choroidal atrophy (2)  Phthisis bulbi (1) | Bradycardia and hypotension (7)  Myelosuppression (6)  Nausea and vomiting (5) | No data | 0 | No data |
